# Prevalence of extracranial carotid artery aneurysms in patients with an intracranial aneurysm

**DOI:** 10.1371/journal.pone.0187479

**Published:** 2017-11-13

**Authors:** V. E. C. Pourier, C. J. H. C. M. van Laarhoven, M. D. I. Vergouwen, G. J. E. Rinkel, Gert J. de Borst

**Affiliations:** 1 Department of Vascular Surgery, University Medical Center Utrecht, Heidelberglaan Utrecht, The Netherlands; 2 Brain Center Rudolf Magnus, Department of Neurology and Neurosurgery, University Medical Center Utrecht, Heidelberglaan Utrecht, the Netherlands; Universitatsklinikum Freiburg, GERMANY

## Abstract

**Background and purpose:**

Aneurysms in various arterial beds have common risk- and genetic factors. Data on the correlation of extracranial carotid artery aneurysms (ECAA) with aneurysms in other vascular territories are lacking. We aimed to investigate the prevalence of ECAA in patients with an intracranial aneurysm (IA).

**Methods:**

We used prospectively collected databases of consecutive patients registered at the University Medical Center Utrecht with an unruptured intracranial aneurysm (UIA) or aneurysmal Subarachnoid hemorrhage (SAH). The medical files of patients included in both databases were screened for availability of radiological reports, imaging of the brain and of the cervical carotid arteries. All available radiological images were then reviewed primarily for the presence of an ECAA and secondarily for an extradural/cavernous carotid or vertebral artery aneurysm. An ECAA was defined as a fusiform dilation ≥150% of the normal internal or common carotid artery or a saccular distention of any size.

**Results:**

We screened 4465 patient records (SAH database n = 3416, UIA database n = 1049), of which 2931 had radiological images of the carotid arteries available. An ECAA was identified in 12/638 patients (1.9%; 95% CI 1.1–3.3) with completely imaged carotid arteries and in 15/2293 patients (0.7%; 95% CI 0.4–1.1) with partially depicted carotid arteries. Seven out of 27 patients had an additional extradural (cavernous or vertebral artery) aneurysm.

**Conclusions:**

This comprehensive study suggests a prevalence for ECAA of approximately 2% of patients with an IA. The rarity of the disease makes screening unnecessary so far. Future registry studies should study the factors associated with IA and ECAA to estimate the prevalence of ECAA in these young patients more accurately.

## Introduction

Extracranial carotid artery aneurysm (ECAA) is rare and accounts for less than 1% of all peripheral artery aneurysms.[1–3] An extracranial carotid artery aneurysm is defined as a dilation of 150% or more of the diameter of the expected normal carotid artery.[4,5] Extracranial carotid artery includes the common carotid artery, the external carotid artery and the internal carotid artery (ICA) till the skull base. ECAAs are mostly incidental findings, commonly asymptomatic, and often identified in the ICA.[4]

Aneurysms in general are known to have common risk- and genetic factors and co-occurrences have been described in other arterial beds.[6] Due to the rarity of ECAAs it is unknown what the incidence, prevalence, association with aneurysms in other vascular territories and best treatment approach is.[7] As far as we know, an analysis for correlation between ECAA and IA has not been systematically performed before.

Datasets of patients with an intracranial aneurysm (IA) are available in our center. Therefore, we had the opportunity to investigate the prevalence of ECAA in patients with an IA.

## Methods

### Patient selection

We performed a retrospective study in two prospectively collected databases of consecutive patients with an IA admitted to or seen at the outpatient clinic of the Department of Neurology and Neurosurgery of the University Medical Center Utrecht, the Netherlands. Approval was obtained from the Institutional Research Ethics Board. Patients were included from 1978 to 2015 in these datasets. All patients provided informed consent for the use of their medical records for research purposes. One database included consecutive patients with subarachnoid hemorrhage (SAH), and one with unruptured intracranial aneurysms (UIA). Duplicates between the datasets were removed (i.e. patients in the UIA database with a ruptured IA during follow up). Patients were also excluded if no IA was present, for example if SAH resulted from trauma, an arteriovenous malformation, dural fistula, dissection without an aneurysm or perimesencephalic hemorrhage.

Two authors (VP, CL) screened the medical records of all patients in both datasets for available radiological imaging. Then, all original imaging (computed tomography angiography (CTA), magnetic resonance angiography (MRA), digital subtraction angiography (DSA), or duplex ultrasound (DUS) was reviewed for the presence of an ECAA. The available CTA evaluation was on 64-section CT scanners and MRA by the use of 1.5Tesla or 3Tesla scanners. Disagreements were discussed with a third independent observer (GB) until a final agreement was reached. Imaged carotid arteries were categorized into completely or partially depicted. Completely imaged carotid arteries were defined as images starting from the aortic arch until and/or beyond the skull base, depicting both the common, internal as well as the external carotid artery. Carotid arteries were considered partially imaged when either side of the internal carotid arteries was completely depicted (i.e. DSA), or when only the distal carotid arteries were depicted until the second cervical vertebra (i.e. CTA Brain).

### Data collection

The diagnosis of an ECAA was determined according to previous radiology reports and by reviewing the available imaging scans of each patient. An ECAA was defined as a dilation of the arterial diameter of ≥150% compared with the normal carotid artery diameter. The side (left versus right, or bilateral), site (common, internal or external carotid artery), shape (saccular, fusiform) and diameter of the aneurysm were retrieved from available reports and by evaluation of the available images by the authors (VP, CL) independently. In addition to the presence of an ECAA, data on other extracranial/extradural cervical arterial aneurysms were collected and: age at presentation, sex, medical history (diabetes, cardiovascular disease, connective tissue disease, polycystic kidney disease), smoking history, medication and clinical presentation.

### Statistical analysis

For continuous variables, we calculated means with standard deviations or medians with ranges. For categorical variables, absolute numbers and/or proportions were calculated with 95% confidence intervals (CI). We calculated the proportion of patients with an ECAA in the complete and partially depicted carotid artery groups.

## Results

### Patients and imaging

**Fig 1:**
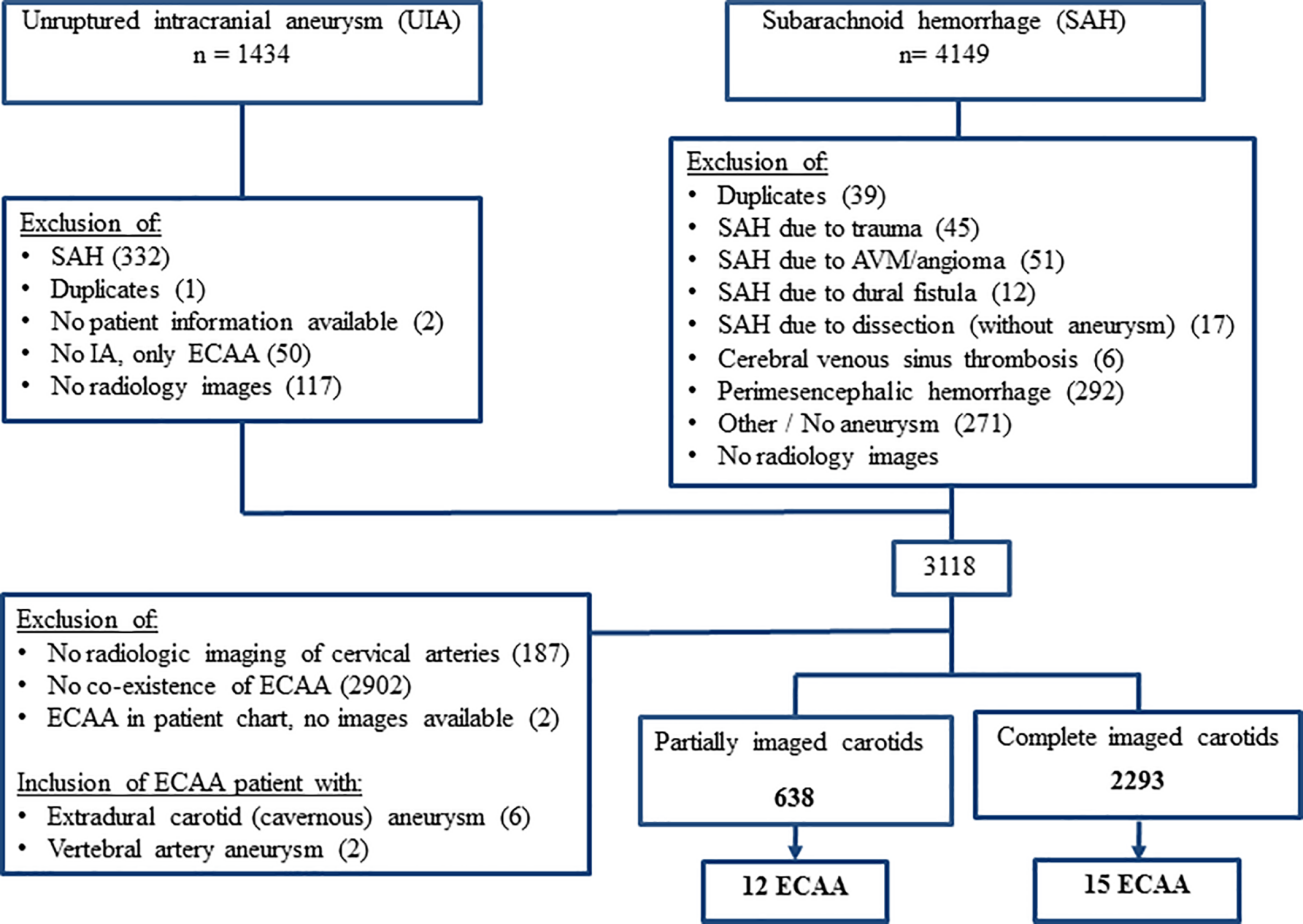
Flowchart of 27 index patients with an IA and ECAA, CA and/or VA. N = number of patients, UIA = non-ruptured intracranial aneurysm, SAH = subarachnoid hemorrhage, AVM = arteriovenous malformation, IA = intracranial aneurysm, ECAA = extracranial carotid aneurysm, CA = extradural cavernous carotid aneurysm, VA = extracranial vertebral aneurysm.

After screening both the databases a total of 3118 (70%) patients remained. Of these patients, 638 (20%) had completely depicted and 2293 (74%) partially depicted carotid arteries (Fig 1). The remaining 187 patients (6%) only had a CT or MRI of the brain without the extracranial cervical arteries being depicted. The available imaging modalities of the cervical arteries were CTA, MRA, DSA, and DUS (Table 1).

**Table 1:** Available radiologic imaging modality of 3118 patients.

			N	PERCENTAGE (%)
**IMAGING OF CERVICAL ARTERIES**				
	No angiography		187	6
	Partially imaged carotid artery		2293	74
		CTA brain	1134	36)
		MRA brain	214	(7)
		DSA brain	941	(30)
		DUS carotids	4	(<1)
	Completely imaged carotid artery		638	(20)
		CTA carotids	310	(10)
		MRA carotids	118	(4)
		DSA carotids	18	(<1)
		DUS carotids	190	(6)
**DIAGNOSIS TIME INTERVAL**				
		Intra- and extradural simultaneously	17	63
		ECAA prior to IA	1	(4)
		IA prior to ECAA	9	(33)
		Time, median (y)	8.9	(3.4–23)

Data in n = number of patients (%). Median, range (y) in years. CTA = computed tomography angiography, MRA = magnetic resonance angiography, DSA = digital subtraction angiography, DSA = digital subtraction angiography, ECAA = extracranial carotid artery aneurysm, IA = intracranial aneurysm

An ECAA was identified in 12 of 638 patients (1.9%; 95% CI 1.1–3.3) with complete imaging of the carotid arteries, and in 15 of 2291 patients (0.7%; 95% CI 0.4–1.1) with partial imaging. Seven of the 27 patients (26%) with an ECAA had an additional extradural aneurysm (cavernous or vertebral artery). In 17 of the 27 (63%) patients the IA and ECAA diagnosis was made simultaneously. In one the ECAA was diagnosed 3.7 years prior to the IA, in nine patients the IA was diagnosed prior to the ECAA because no imaging of the carotids was performed or available before.

### Patient characteristics

Characteristics of the patients with an ECAA are presented in Table 2. The median age was 55 years (31–85). Fifteen patients (56%) were women. Of all the patients with an ECAA, 24 (89%) had no symptoms associated with the ECAA.

**Table 2:** Characteristics of 27 patients with an ECAA.

			N	PERCENTAGE (%)
**WOMEN**			15	56
**MEDICAL HISTORY**				
	Diabetes		1	4
	Vascular		12	44
	TIA/Ischemic stroke		5	19
	Connective tissue disorder		2	7
	Trauma in cervical region		0	0
	Polycystic kidney disease		1	4
**SMOKING HISTORY**				
	Never		7	26
	Quit smoking		7	26
	Current smoker		9	33
	Unknown		4	15
**MEDICATION USE**				
	None		12	4
	Anti-hypertensive drugs		9	33
	Anti-thrombotic drugs		5	19
		Mono-therapy	4	15
		Multiple-therapy	1	4
	Lipid-lowering drugs		3	11
	Unknown		3	11
**FAMILY HISTORY**				
	Aneurysm		2	7
	Cardiovascular		8	30
	CTD		0	0
	PCKD		1	4
	Unknown		8	27
**CLINICAL PRESENTATION**				
	Asymptomatic		24	89
	Ipsilateral ischemic stroke		1	4
	Horner’s syndrome		1	4
	Local pain		1	4

N = Number. of patients. ECAA = extracranial carotid aneurysm. CTD = connective tissue disease, PCKD = Polycystic kidney disease

### Aneurysm characteristics

Aneurysm characteristics are summarized in Table 3. All ECAAs were located in the internal carotid artery. The shape of the ECAA was saccular (n = 17) or fusiform (n = 17). The median size of the saccular ECAA was 8.0 mm (range 4–13 mm) and for fusiform ECAA 9.0 mm (range 6–12.5 mm). The etiology was mainly unknown, in five cases it was due to dissection, based on radiology images (visible flap) and in one patient due to connective tissue disorder, namely Ehlers-Danlos type IV.

**Table 3:** Aneurysm characteristics.

		IA, N	PERCENTAGE (%)	ECAA, N	PERCENTAGE (%)
**RUPTURED**		17	63	0	
**SIDE**					
	Left	6	16	8	30
	Right	10	26	12	44
	Bilateral	11	58	7	26
**SITE**					
	CCA			0	
	ECA			0	
	ICA			34	100
	Extradural cavernous carotid artery			6 ^b^	
	Extracranial vertebral artery			2 ^b^	
**SHAPE**					
	Saccular			17	50
	Fusiform			17	50
**SIZE IN MM, MEDIAN (RANGE)**					
	Saccular			8.4 (4–13)	
	Fusiform			9.2 (6–12.5)	
**PRESUMED ETIOLOGY**					
	Dissection			5	15
	CTD			2	6
	Not reported			28	79

^b^ Patients with additional extradural cavernous carotid and vertebral artery aneurysm, not included in analysis.

Data in: n = number of aneurysms. IA = intracranial aneurysm, ECAA = extracranial carotid aneurysm, CCA = common carotid artery, ECA = external carotid artery, ICA = internal carotid artery, CTD = connective tissue disorder.

## Discussion

The present study shows that approximately 2% of the patients with an IA and completely imaged carotid arteries has an ECAA. No studies systematically investigated the prevalence of IA and ECAA before. [6] This prevalence could be an underestimation due to heterogeneity of the imaging modalities. In some patients DUS was used, but this modality is operator dependent and it cannot accurately detect distal extracranial carotid aneurysms. [8]

It remains unclear if IAs and ECAAs share the same etiology in the same patients. IAs are mostly saccular shaped,[9] while ECAAs have so far been described to be almost equally divided fusiform and saccular shaped.[4] Also, the wall structure of intracranial and extracranial vessels differs, which may indicate a different pathophysiology.[10] Atherosclerosis and dissection have been described as a main cause of ECAAs, whereas IA have been described to have changes of atheroslerosis.[4,6,11–13] The next step in research to be taken is to analyze large prospective imaging databases on patients with proven extracranial and intracranial carotid artery dissection to study the long term course and co-development of ECAA and IA along the dissection track. The development of extradural aneurysms should be studied in the same analysis.

All identified ECAAs were located in the internal carotid artery; these would be classified as Attigah type I—III, these are aneurysms that involve the ICA and are located above the bulb.[14] This is in accordance to our not yet published prospective clinical data from an ongoing web-based registry (www.carotidaneurysmregistry.com).[13,15] Within this registry patients with any extracranial carotid artery aneurysm can be included, independent of the type of treatment recommended and initiated, ranging from conservative treatment with follow-up to endovascular intervention or open surgical exclusion. With the data included in the registry, we can investigate in the future whether there is a correlation between ECAA and IA.

## Conclusion

This is the first prevalence study of ECAA in patients with an IA. In this single-center, retrospective study we found a prevalence of 1.9%. This prevalence indicates the possible rarity of the disease for which screening has not been indicated so far. However, the sparse knowledge on optimal work-up of ECAA is in contrast with the interest in management and long-term clinical outcome of relatively young patients, if left untreated. Future registry studies may elucidate the factors leading to co-existence of both IA and ECAA and estimate the prevalence of ECAA in patients with an IA more accurately.
